# The impact of treatment on the psychological burden of mothers of children with chronic hepatitis C virus infection: a multicenter, questionnaire survey

**DOI:** 10.1038/s41598-022-25519-1

**Published:** 2022-12-21

**Authors:** Tomoya Fukuoka, Kazuhiko Bessho, Satoyo Hosono, Daiki Abukawa, Tatsuki Mizuochi, Koichi Ito, Jun Murakami, Hideo Tanaka, Yoko Miyoshi, Tomoko Takano, Hitoshi Tajiri

**Affiliations:** 1 Department of Pediatrics, Osaka University Graduate School of Medicine, Osaka, Japan; 2 Division of Cancer Screening Assessment and Management, National Cancer Center Japan Institute for Cancer Control, Tokyo, Japan; 3 Division of General Pediatrics and Gastroenterology, Miyagi Children’s Hospital, Miyagi, Japan; 4 Department of Pediatrics and Child Health, Kurume University School of Medicine, Fukuoka, Japan; 5 Department of Pediatrics and Neonatology, Nagoya City University Graduate School of Medical Sciences, Nagoya, Japan; 6 Division of Pediatrics and Perinatology, Faculty of Medicine, Tottori University, Tottori, Japan; 7 Osaka Prefecture Fujiidera Public Health Center, Osaka, Japan; 8 Department of Pediatrics, Osaka General Medical Center, Osaka, Japan; 9 Department of Pediatrics, Faculty of Medicine Hospital, Kinki University, Osaka, Japan

**Keywords:** Psychology, Gastroenterology

## Abstract

Mothers of children with chronic hepatitis C virus (HCV) infection experience anxiety about the health of their children. In this study we assessed an impact of treating children with chronic HCV infection on the psychological burden of their mothers. This was a multicenter, questionnaire survey conducted at six institutions in Japan. A newly-developed questionnaire for this study was used to assess changes in the mothers’ various concerns regarding HCV infection and thoughts about their child’s HCV infection. Responses at the time of diagnosis and at the time of the survey were compared between mothers of children who had received treatment and those without treatment. Responses were received from 36 of 37 eligible mothers (11 and 25, non-treatment and treatment groups, respectively). All children in treatment group had successfully eliminated the virus. Mothers in both groups were psychologically stressed in various ways, including concern about their child’s health in the present and future at the time of diagnosis, concern about school, employment, and marriage, concern about the behavior of others towards them and infecting others with HCV, and feelings of guilt regarding their child. These concerns were significantly lower in the present compared to at the time of diagnosis in treatment group, and the rate of decrease was significantly higher in treatment group compared to non-treatment group. Successful treatment greatly reduced mothers’ concerns about their children’s HCV infection, indicating that treatment during childhood is beneficial from the perspective of the mothers’ psychological burden.

## Introduction

Chronic hepatitis C is a major cause of chronic liver disease. Continued inflammation from the hepatitis C virus (HCV) leads to hepatic fibrosis and progresses to cirrhosis and hepatocellular carcinoma^1,2^. It is estimated that 56.8 million people worldwide have viremic HCV infection^3^. The global estimate for viremic prevalence in the pediatric population aged 0–18 years was 0.13%, which corresponds to 3.26 million children in 2018^4^. Hepatitis progresses slowly in most children, but it can lead to hepatic fibrosis or cirrhosis in some cases, requiring liver transplantation^5,6^. Children with chronic HCV infection also show poorer mental health, cognitive function, and quality of life than healthy children^7–9^. Treatment of chronic hepatitis C was based on interferon (IFN) for many years, but direct-acting antivirals (DAAs) have recently emerged, and studies have confirmed that various DAA regimens are both safe and effective for children^10–14^. To treat chronic hepatitis C, the United States Food and Drug Administration (FDA) has approved sofosbuvir/ledipasvir for adolescents aged 12–17 years^10^, for children aged 6–11 years^11^ and for children aged 3 to < 6 years^12^. Glecaprevir/pibrentasvir was also approved for children aged 12 years and older^13^ and for children aged 3–11 years^14^.

The main route of HCV Infection in children was previously through blood transfusions, but such infections have been reduced by the introduction of HCV antibody screening of blood donors, and the current main route of infection is vertical transmission from the mother^15^. There is no established method for preventing vertical transmission, and about 10% of children born to HCV carrier mothers perinatally contracted HCV in Japan^16^. Caregivers of children with HCV have been found to have high concern and worry about the children’s current and future physical health, emotional well-being, and general behavior^7,17^. Moreover, among caregivers, the psychological burden of the mothers who transmitted HCV to their child is inestimable. Although it is thought that such concern could be managed by eliminating the virus from the child through treatment, no studies have assessed the impact of treatment on this burden.

Therefore, in this study, the concerns of mothers of children with HCV irrespective of history of treatment were compared using a questionnaire developed originally for the study, and the impact of treatment on the mothers’ concerns about their children and thoughts about HCV infection in children was evaluated.

## Methods

### Interview survey of a small group of caregivers

At first, to clarify psychology of caregivers of HCV-infected children, we conducted an interview survey of five parents (all mothers) whose children were visiting the Pediatrics department of the Osaka General Medical Center. With the parent’s consent recordings also were taken at the time of the interview to secure accuracy. The 10 items to be interviewed are as follows: (1) baseline characteristics of parents (age, occupation, educational background, their medical history, family history, family relationships, etc.), (2) baseline characteristics of the child (age, educational background, current health condition, age at diagnosis and at treatment, how to explain the illness to the child, treatment content, treatment period, etc.), (3) knowledge of HCV infection and mother-to-child transmission (correlation between high level of understanding and psychological burden, are there any items that are misunderstood, etc.), (4) explanation from the doctor in charge (Is it possible to understand the content of the explanation, what kind of situation it was explained in, and how it felt?), (5) disliked or troubled experiences, related to HCV infection for the guardians, (6) ask for keywords related to feelings of guilt, (7) current anxiety and troubles, (8) future anxiety (advancement, employment, marriage, progress from chronic hepatitis to liver cirrhosis and hepatic cancer), (9) anxiety about recurrence, good and bad treatment, changes in feelings, etc. (only for those who received treatment. Did the psychological burden and feelings of guilt improve?), (10) others.

The interview narratives of the five mothers were summarized to clarify the psychological burden of parents and the needs and significance of curative treatment in childhood.

### Creation of questionnaire survey form and multicenter questionnaire survey

We prepared a versatile questionnaire survey form based on the analysis of the results obtained from the above-mentioned interview survey. The form basically includes items on concerns about HCV infection in their child and on thoughts about pediatric HCV infection (Table S1). To assess the items on concerns, responses were given on an 11-point scale from 0 (no concern) to 10 (strong concern).

From July 2018 to May 2019, the questionnaire survey forms were administered to mothers of children with vertically-transmitted chronic HCV infection visiting one of six facilities in Japan (Miyagi Children’s Hospital, Nagoya City University Hospital, Osaka University Hospital, Osaka General Medical Center, Tottori University Hospital, and Kurume University Hospital). They were asked about baseline characteristics of themselves and their child, as well as their concerns and thoughts about pediatric HCV infection at the time of diagnosis and in the present. Scores were compared at the time of diagnosis and the time of completing the questionnaire (the present) between mothers of children who had received treatment (Treatment group) and those without treatment (No treatment group), and the rate of change in scores was compared between groups. For questions related to concerns about school and concerns about future participation in society (employment), mothers whose children were already employed were asked to respond for the period immediately following the end of treatment instead of the current situation at the time of completing the questionnaire. For questions related to thoughts about pediatric HCV infection, responses at the time of diagnosis were chosen from ‘strongly disagree,’ ‘disagree,’ ‘neither agree nor disagree,’ ‘agree,’ and ‘strongly agree’ for each question. The rates of mothers who chose ‘strongly agree’ or ‘agree’, were compared between groups.

### Statistics

EZR (Jichi Medical University Saitama Medical Center)^18^ was used for all statistical analyses, with a significance level of p < 0.05. Kolmogorov–Smirnov tests were used to verify the normality of continuous variables. When comparing the two groups, homogeneity of variance was tested with *F* tests, and the groups were compared with Student’s *t* tests or Welch’s *t* tests. When the result was not a normal distribution, the groups were compared with the Wilcoxon rank-sum test. Wilcoxon signed-rank tests were used for group comparisons of scores for questions related to concerns about the children’s HCV infection at the time of diagnosis and in the present. Fisher’s exact test was used to compare rates. The Bonferroni correction was applied for multiple comparisons.

### Ethical consideration

Before the study, written, informed consent was obtained from the mothers of children with chronic HCV infection. The study protocol complies with the ethical guidelines of the Declaration of Helsinki of 1975 (2004 revision) and was approved by the Ethics Committee of Osaka General Medical Center and also by each of the remaining five institution’s ethics committee. All research was performed in accordance with relevant guidelines/regulations.

## Results

### Sample

A total of 37 mothers were surveyed, but one was excluded for insufficient responses, leaving 36 mothers in the analysis. There were 25 mothers in the treatment group and 11 in the non-treatment group. The mothers’ baseline characteristics are shown in Table 1. All mothers had chronic HCV infection. The mothers were significantly older in the treatment group (49.5 ± 5.5 years vs 40.5 ± 4.6 years, p < 0.001), but no differences were observed in age at diagnosis, parity, rate of relatives with cirrhosis, or rate with hepatocellular carcinoma. Regarding the mothers’ treatment, no differences were observed in treatment history, therapeutic drug treatment, or rate with moderate to severe side effects, and all mothers except one in the non-treatment group had successfully eliminated the virus. Table 2 shows the baseline characteristics of the children. The children were significantly older in the treatment group (16.6 ± 4.6 years vs 8.1 ± 2.6 years, p < 0.01), but no differences were observed in sex, age at diagnosis, or rate with illnesses other than HCV infection. Four children in the treatment group and none in the non-treatment group were employed. The route of infection was via the mother in all cases of the both groups. In the treatment group, HCV treatment was received at an average of 10.9 years, and 40% of children experienced moderate to severe side effects. All the children in this group successfully eliminated the virus, 88% with the first treatment.

**Table 1:** Sociodemographic and medical characteristics in the mothers of children with HCV infection.

	Non-treatment group (n = 11)	Treatment group (n = 25)	p value
Age at this study, y, mean ± SD	40.5 ± 4.6	49.5 ± 5.5	p < 0.001
**Final academic background (%)**			n.s.
Junior high school	1 (8.3)	2 (8.0)	
High school	3 (27.3)	10 (40.0)	
Career college	1 (8.3)	3 (12.0)	
University	5 (41.7)	10 (40.0)	
Others	1 (8.3)	0 (0.0)	
Number of deliverly, mean ± SD	1.9 ± 0.5	2.4 ± 0.8	n.s.
Cirrhosis in relatives (%)	2 (18.1)	2 (8.0)	n.s.
HCC in relatives (%)	1 (8.3)	1 (4.0)	n.s.
Mother’s HCV infection (%)	11 (100)	25 (100)	n.s.
Age at infection detected, y, mean ± SD	26.8 ± 4.9	27.4 ± 5.5	n.s.
Experience of HCV treatment (%)	10 (90.9)	20 (80.0)	n.s.
**Therapeutic agents (%)**			n.s.
IFN	1 (10.0)	2 (10.0)	
Peg-IFN + RBV	3 (30.0)	11 (55.0)	
DAA	7 (70.0)	14 (70.0)	
Moderate or severe adverse events (%)	2 (20.0)	9 (45.0)	n.s.
Viral clearance (%)	9 (90.0)	20 (100)	n.s.

*IFN* interferon, *Peg-IFN* Peg-interferon, *RBV* ribavirin, *DAA* direct acting antivial.

**Table 2:** Characteristics of children with HCV infection.

	Non-treatment group (n = 11)	Treatment group (n = 25)	p-value
Male, n, (%)	4 (36.3)	13 (52.0)	n.s.
Age at this study, y, mean ± SD	8.1 ± 2.6	16.6 ± 4.6	p < 0.01
Past medical history other than hepatitis C, n (%)	1 (9.1)	3 (12.0)	n.s.
Age at infection detected, y, median (interquadrant range)	0 (0–1)	0 (0–1.25)	n.s.
**Transmission mode (%)**			
Vertical/perinatal	11 (100)	25 (100)	n.s.
Age at initial treatment, y, mean ± SD		10.9 ± 4.3	
**Number of treatment (%)**			
0	11 (100)	0 (0)	
1	0 (0)	22 (88.0)	
2	0 (0)	2 (8.0)	
3	0 (0)	1 (4.0)	
**Therapeutic agents (%)**			
IFN		6 (24.0)	
Peg-IFN + RBV		12 (48.0)	
Peg-IFN + RBV + SMV		2 (8.0)	
DAA		8 (32.0)	
Moderate or severe adverse events (%)		10 (40.0)	
Viral clearance (%)		25 (100)	

*IFN* interferon, *Peg-IFN* Peg-interferon, *RBV* ribavirin, *SMV* simeprevir, *DAA* direct acting antivial.

### Mothers’ concerns about HCV infection in their children

**Figure 1:**
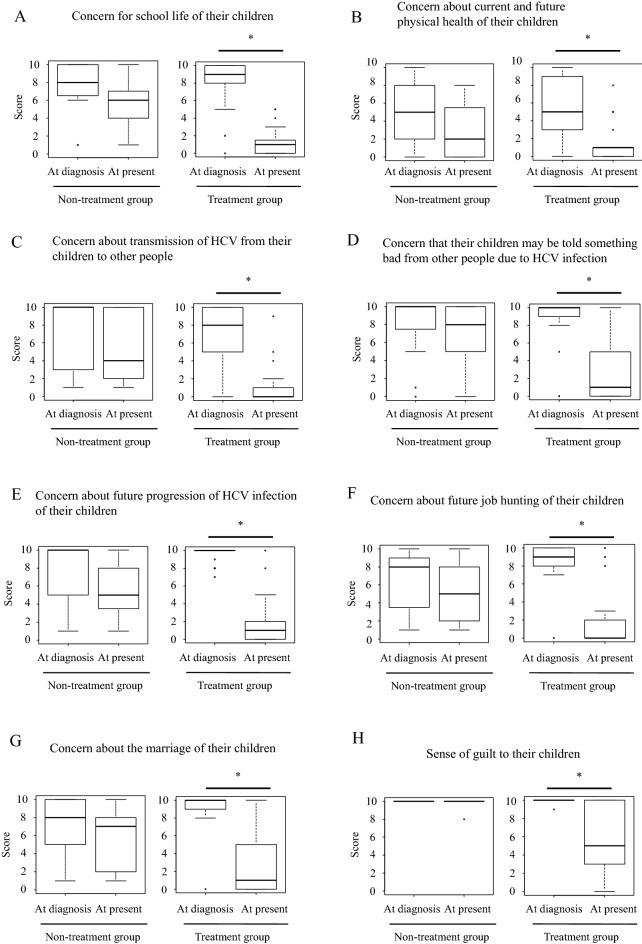
Box and whisker plot of the scores at the time of diagnosis and at the present for the indicated questions related to mothers’ concerns about HCV infection in their children in Group A and B. *Represents with p < 0.05.

**Figure 2:**
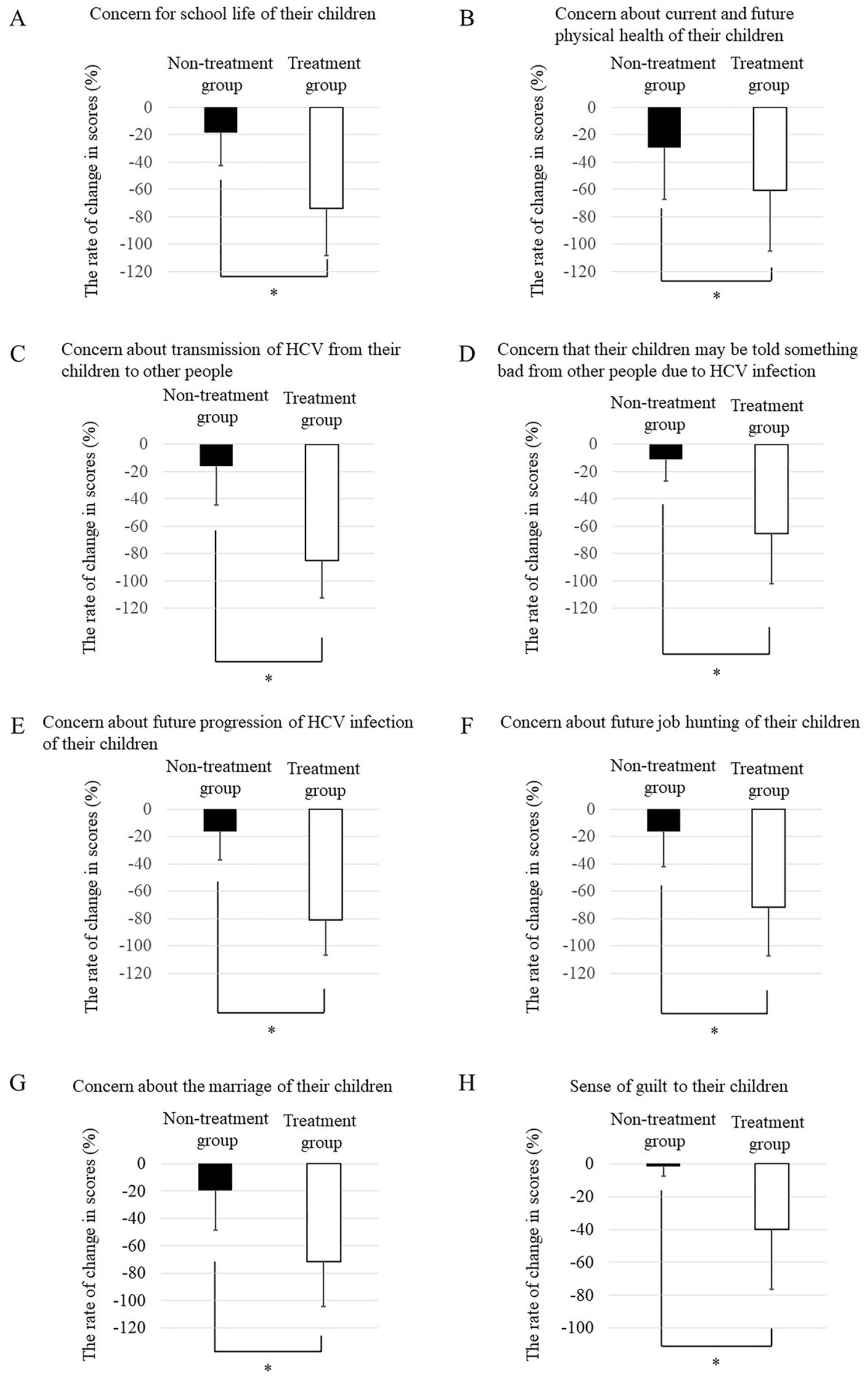
The rate of change in scores from the time of diagnosis to the present for the indicated questions related to mothers’ concerns about HCV infection in their children in Group A and B. *Represents with p < 0.05.

Both groups scored high on almost all questions related to concerns about HCV infection in their children at the time of diagnosis (Table S2). In the non-treatment group, scores were significantly lower in the present than at the time of diagnosis for three questions (concerns about school, concern that the child’s HCV could progress further in the future, and concerns about marriage). In contrast, scores for all questions were significantly lower in the present than at the time of diagnosis in the treatment group (Fig. 1). Almost all mothers scored 10 points (highest score) on feelings of guilt regarding their child at the time of diagnosis of vertical transmission of HCV infection. That score was lower in the present in the treatment group, but not in the non-treatment group. Next, the intergroup comparison of the rate of change in scores showed a larger rate of decrease in scores in the treatment group than in the non-treatment group for all questions (Fig. 2).

### Mother’s thoughts about pediatric hepatitis C virus infection

Table 3 and Table S3 show the responses by mothers of children with chronic HCV infection in the treatment group and the non-treatment group to questions related to their thoughts about pediatric HCV infection at the time of diagnosis. Although the rate of mothers who responded with ‘agree’ or ‘strongly agree’ to the statement ‘Chronic hepatitis C in children leads to cirrhosis and HCC in the future’ tended to be higher in the treatment group than in non-treatment group (92.0% vs 66.7%, p = 0.0728), no significant differences in responses between two groups were observed for any questions.

**Table 3:** The rate of mothers who answered ‘agree’ or ‘strongly agree’ to the questions related to the thoughts about pediatric hepatitis C virus infection.

	Non-treatment group	Treatment group	p-value
1. Hepatitis C virus infection is rare in children, n (%)	7 (63.6)	15 (60.0)	n.s.
2. Pediatric hepatitis C virus infection is severe disease, n (%)	6 (54.5)	18 (72.0)	n.s.
3. Chronic hepatitis C in children leads to cirrhosis and HCC in the future, n (%)	8 (72.7)	23 (92.0)	n.s.
4. Treatment of hepatitis C virus infection is a heavy burden in children, n (%)	8 (72.7)	20 (80.0)	n.s.
5. HCV infection in children is easily cured with treatment, n (%)	3 (27.3)	12 (48.0)	n.s.
6. Hepatitis C virus has high infectivity, n (%)	2 (18.2)	10 (40.0)	n.s.
7. Hepatitis C virus infection can be prevented under appropriate management, n (%)	9 (81.8)	20 (80.0)	n.s.

## Discussion

This is the first study to assess the effects of treatment both on psychological stress that mothers of children with chronic HCV infection feel about their children’s HCV infection and on their thoughts about their children’s HCV infection.

Our results show that the mothers of children with chronic HCV infection were psychologically stressed in various ways; including concern about their child’s health in the present and future from the time of diagnosis, concern about school, employment, and marriage, and concern about how other people might behave towards them and infecting others with HCV. Moreover, the mothers had quite strong feelings of guilt for their child. Our results support the finding by Nydegger et al.^17^ that parents of children with chronic HCV infection worry about their child’s health and that 70–80% worry about them being treated differently by friends and teachers because of their HCV infection. This result highlights the importance of considering the mothers’ psychological burden when providing treatment to children with HCV.

In the treatment group, all the children had successfully eliminated the virus. Within this context, scores decreased significantly for all questions in this group, with significantly higher rates of decrease compared to the non-treatment group in all questions. This suggests that the various types of psychological stress caused by their children’s HCV infection may have lessened in mothers in this group due to the success of treatment. Among the psychological burden of the mothers, our results show that how strong the sense of guilt the mother had against the fact that she had transmitted the virus to her child during the perinatal period, and the feeling of guilt was not alleviated in the untreated group. However, the median score for the sense of guilt was reduced by half in the group that was treated with successful clearance of the virus. The feeling of guilt is a feeling that is very socially and culturally sympathetic in some Asian countries including Japan as well as western countries in the Christian sphere. Our study shows novel findings that the treatment contributed to alleviate this burden in mothers besides clearance of the virus.

Pediatric HCV infection advances slowly, and few cases progress to cirrhosis or hepatocellular carcinoma in childhood. For this reason, when treatment was still centered on IFN, which has been shown to have side effects such as impaired growth, there was debate over whether children with chronic HCV infection should be treated while young^19^. Studies then proved that DAA medications were both safe and effective for chronic hepatitis C in children^9–14^, and various guidelines and position papers now recommend this treatment for chronic hepatitis C in children aged 12 years and over for whom DAA treatment has been approved^20–24^. Treating chronic hepatitis C in childhood offers potential benefits. For one, it may reduce the risk of developing cirrhosis or hepatocellular carcinoma from chronic hepatitis and could improve quality of life and reduce cognitive impairment associated with chronic HCV infection^8^. Furthermore, they demonstrated that DAA treatment improved quality of life of caregivers as well as that of children with HCV^9^. Second, treatment in childhood may lower medical expenses associated with chronic hepatitis^25,26^. In addition to these benefits, the present study shows that treatment in childhood may offer the benefit of reducing the psychological stress on mothers. Although the eligible age for treatment is expected to decrease, and earlier treatment may be preferable from the perspective of the psychological burden on the mother, the results of clinical trials of DAA therapy currently being conducted on younger children and the possibility of spontaneous cure must be considered when determining the age to start treatment.

HCV-infected adult patients showed subtle cognitive defects, possibly linked to direct brain involvement by the virus^27^. DAA treatment improves HRQL not only in HCV-infected adolescents but also in caregivers during treatment and is maintained after SVR^9^. We speculate that the reported improvements by DAA treatment in HCV-infected patients may be related to SVR rather than a direct effect of DAAs on cognitive impairment in the brain. Our study suggests that improvement in the caregiver's anxiety score immediately after the end of treatment may have been influenced by the improvement in their children's condition. A placebo-controlled trial is needed to clarify this point.

We hypothesized that the mothers in the treatment group might be more serious about HCV infection in their children at the time of diagnosis, which leads to the treatment of their children’s HCV infection. Therefore, we investigated the difference in the mothers’ thoughts about pediatric HCV infection between treatment group and non-treatment group. The rate of mothers who responded with ‘agree’ or ‘strongly agree’ to the statements ‘Pediatric hepatitis C virus infection is severe disease’ and ‘Chronic hepatitis C in children leads to cirrhosis and HCC in the future’ was slightly higher in the treatment group, however, we found no significant differences between two groups in the responses to these questions as with other questions. This result suggests that the underlying characteristics of mothers in the thoughts about HCV infection in children are quite similar in these two groups, however, retrospective design and relatively small sample size may limit this result.

The age of the children at the time of completing the survey in the present study was significantly older in the treatment group. However, no other group differences were observed in the baseline characteristics of the mothers or the children. In addition, the present age of the children in the non-treatment group had not yet reached the mean age at the start of treatment in the treatment group, and chronic hepatitis C is not aggressively treated early in childhood in most cases. Thus, children in the non-treatment group will likely receive treatment in the future. The characteristics of the groups may therefore be considered similar.

This study has several limitations. Since the mothers were required to remember concern and feelings of guilt at diagnosis of HCV infection for their child, recall bias on the assessment of changing in those factors might occur. It should be also noted that recall bias is more likely to make a significant difference in the treatment group. However, since the median of these factors in the treatment group were almost equal with those in the non-treatment group, the impact of the recall bias between non-treatment group and treatment group would not be so different. Of note, the age of the children differed between the two groups, and the possibility remains that the children’s age or duration of illness affected concerns about pediatric HCV infection. Finally, this study ideally includes more respondents because a small number of subjects limit the power to make solid conclusions. A prospective study with more cases is needed to resolve these limitations of this study.

The results of the present study demonstrate that mothers of children with chronic HCV infection have various concerns about the children, such as their health, the behavior of others towards them, and their future, as well as feelings of guilt toward them, and these can be reduced by treatment. Successful treatment during childhood would therefore be beneficial from the perspective of the psychological burden on the mothers. Further research including prospective studies is warranted to confirm the results of this study.

## Supplementary Information

Supplementary Table S1.

Supplementary Table S2.

Supplementary Table S3.

## Data Availability

The datasets during and/or analyzed during the current study are available from the corresponding author on reasonable request.

## References

[1] Kiyosawa K, Sodeyama T, Tanaka E (1990). Interrelationship of blood transfusion, non-A, non-B hepatitis and hepatocellular carcinoma: Analysis by detection of antibody to hepatitis C virus. Hepatology.

[2] El Khoury AC, Vietri J, Prajapati G (2012). The burden of untreated hepatitis C virus infection: A US patients' perspective. Dig. Dis. Sci..

[3] Polaris Observatory HCV Collaborators (2022). Global change in hepatitis C virus prevalence and cascade of care between 2015 and 2020: A modelling study. Lancet Gastroenterol. Hepatol..

[4] Schmelzer J, Dugan E, Blach S (2020). Global prevalence of hepatitis C virus in children in 2018: A modelling study. Lancet Gastroenterol. Hepatol..

[5] Bortolotti F, Verucchi G, Camma C (2008). Long-term course of chronic hepatitis C in children: From viral clearance to end-stage liver disease. Gastroenterology.

[6] Squires JE, Balistreri WF (2017). Hepatitis C virus infection in children and adolescents. Hepatol. Commun..

[7] Rodrigue JR, Balistreri W, Haber B (2009). Impact of hepatitis C virus infection on children and their caregivers: Quality of life, cognitive, and emotional outcomes. J. Pediatr. Gastroenterol. Nutr..

[8] Younossi Z, Park H, Henry L, Adeyemi A, Stepanova M (2016). Extrahepatic manifestations of hepatitis C: A meta-analysis of prevalence, quality of life, and economic burden. Gastroenterology.

[9] Younossi ZM, Stepanova M, Balistreri W, Schwarz K, Murray KF, Rosenthal P (2018). Health-related quality of life in adolescent patients with hepatitis C genotype 1 treated with sofosbuvir and ledipasvir. J. Pediatr. Gastroenterol. Nutr..

[10] Balistreri WF, Murray KF, Rosenthal P (2017). The safety and effectiveness of ledipasvir–sofosbuvir in adolescents 12–17 years old with hepatitis C virus genotype 1 infection. Hepatology.

[11] Murray KF, Balistreri WF, Bansal S (2018). Safety and efficacy of ledipasvir–sofosbuvir with or without ribavirin for chronic hepatitis C in children ages 6–11. Hepatology.

[12] Schwarz KB, Rosenthal P, Murray KF (2020). Ledipasvir–sofosbuvir for 12 weeks in children 3 to <6 years old with chronic hepatitis C. Hepatology.

[13] Jonas MM, Squires RH, Rhee SM (2020). Pharmacokinetics, safety, and efficacy of glecaprevir/pibrentasvir in adolescents with chronic hepatitis C virus: Part 1 of the DORA Study. Hepatology.

[14] Jonas MM, Rhee S, Kelly DA (2021). Pharmacokinetics, safety, and efficacy of glecaprevir/pibrentasvir in children with chronic HCV: Part 2 of the DORA study. Hepatology.

[15] Indolfi G, Azzari C, Resti M (2013). Perinatal transmission of hepatitis C virus. J. Pediatr..

[16] Okamoto M, Nagata I, Murakami J (2000). Prospective reevaluation of risk factors in mother-to-child transmission of hepatitis C virus: High virus load, vaginal delivery, and negative anti-NS4 antibody. J. Infect. Dis..

[17] Nydegger A, Srivastava A, Wake M, Smith AL, Hardikar W (2008). Health-related quality of life in children with hepatitis C acquired in the first year of life. J. Gastroenterol. Hepatol..

[18] Kanda Y (2013). Investigation of the freely available easy-to-use software 'EZR' for medical statistics. Bone Marrow Transplant..

[19] Mack CL, Gonzalez-Peralta RP, Gupta N (2012). NASPGHAN practice guidelines: Diagnosis and management of hepatitis C infection in infants, children, and adolescents. J. Pediatr. Gastroenterol. Nutr..

[20] Indolfi G, Hierro L, Dezsofi A (2018). Treatment of chronic hepatitis c virus infection in children: A position paper by the Hepatology Committee of European Society of Paediatric Gastroenterology, Hepatology and Nutrition. J. Pediatr. Gastroenterol. Nutr..

[21] European Association for the Study of Liver (2018). EASL recommendations on treatment of hepatitis C 2018. J. Hepatol..

[22] Lagging M, Wejstal R, Duberg AS, Aleman S, Weiland O, Westin J (2018). Treatment of hepatitis C virus infection for adults and children: Updated Swedish consensus guidelines 2017. Infect. Dis. (Lond)..

[23] AASLD-IDSA HCV Guidance Panel (2018). guidance 2018 update: AASLD-IDSA recommendations for testing, managing, and treating hepatitis C virus infection. Clin. Infect. Dis..

[24] World Health Organization (2018). WHO Guidelines Approved by the Guidelines Review Committee. Guidelines for the Care and Treatment of Persons Diagnosed with Chronic Hepatitis C Virus Infection.

[25] Sinha M, Das A (2000). Cost effectiveness analysis of different strategies of management of chronic hepatitis C infection in children. Pediatr. Infect. Dis. J..

[26] Nguyen J, Barritt AST, Jhaveri R (2019). Cost effectiveness of early treatment with direct-acting antiviral therapy in adolescent patients with hepatitis C virus infection. J. Pediatr..

[27] Vaghi G, Gori B, Strigaro G (2020). Direct antivirals and cognitive impairment in hepatitis C: A clinical-neurophysiologic study. J. Neurovirol..

