# Supplementary figures and images for: The impact of treatment on the psychological burden of mothers of children with chronic hepatitis C virus infection: a multicenter, questionnaire survey
Source: Sci Rep. 2022 Dec 21;12:22116. doi: 10.1038/s41598-022-25519-1 (PMC9772351; doi:10.1038/s41598-022-25519-1)

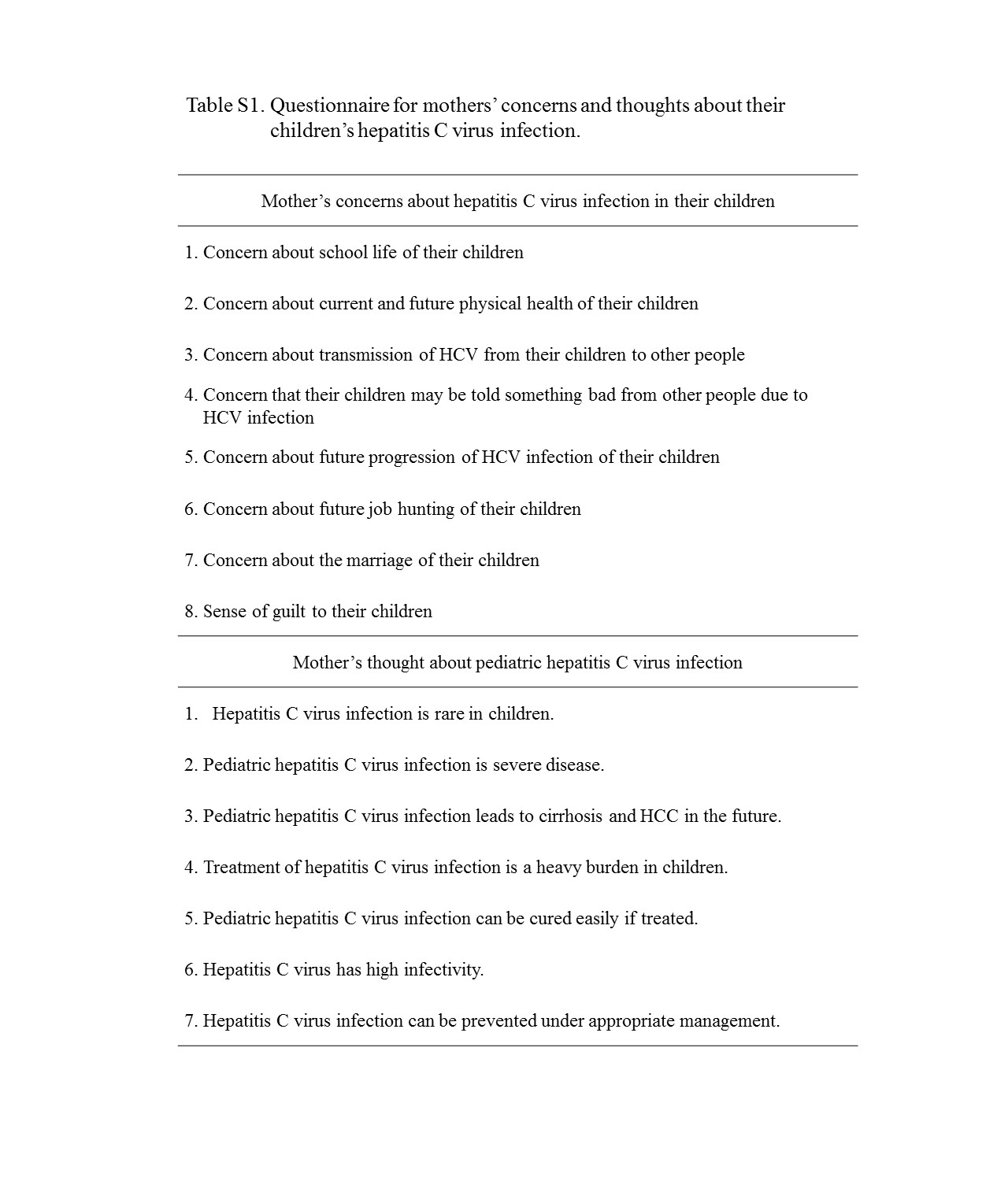

Supplement: Supplementary file 1 — Supplementary Table S1. [file 41598_2022_25519_MOESM1_ESM.jpg]

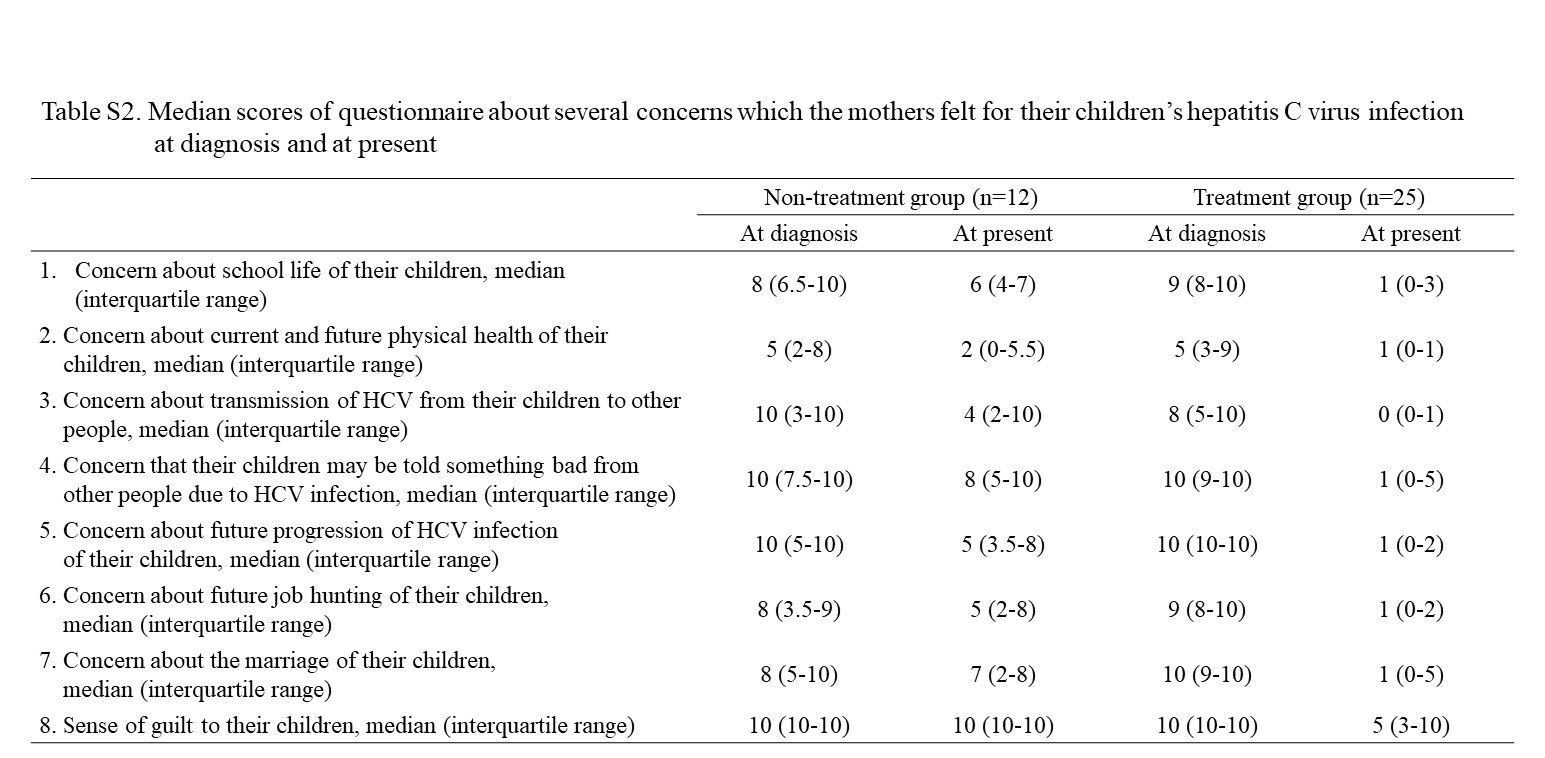

Supplement: Supplementary file 2 — Supplementary Table S2. [file 41598_2022_25519_MOESM2_ESM.jpg]

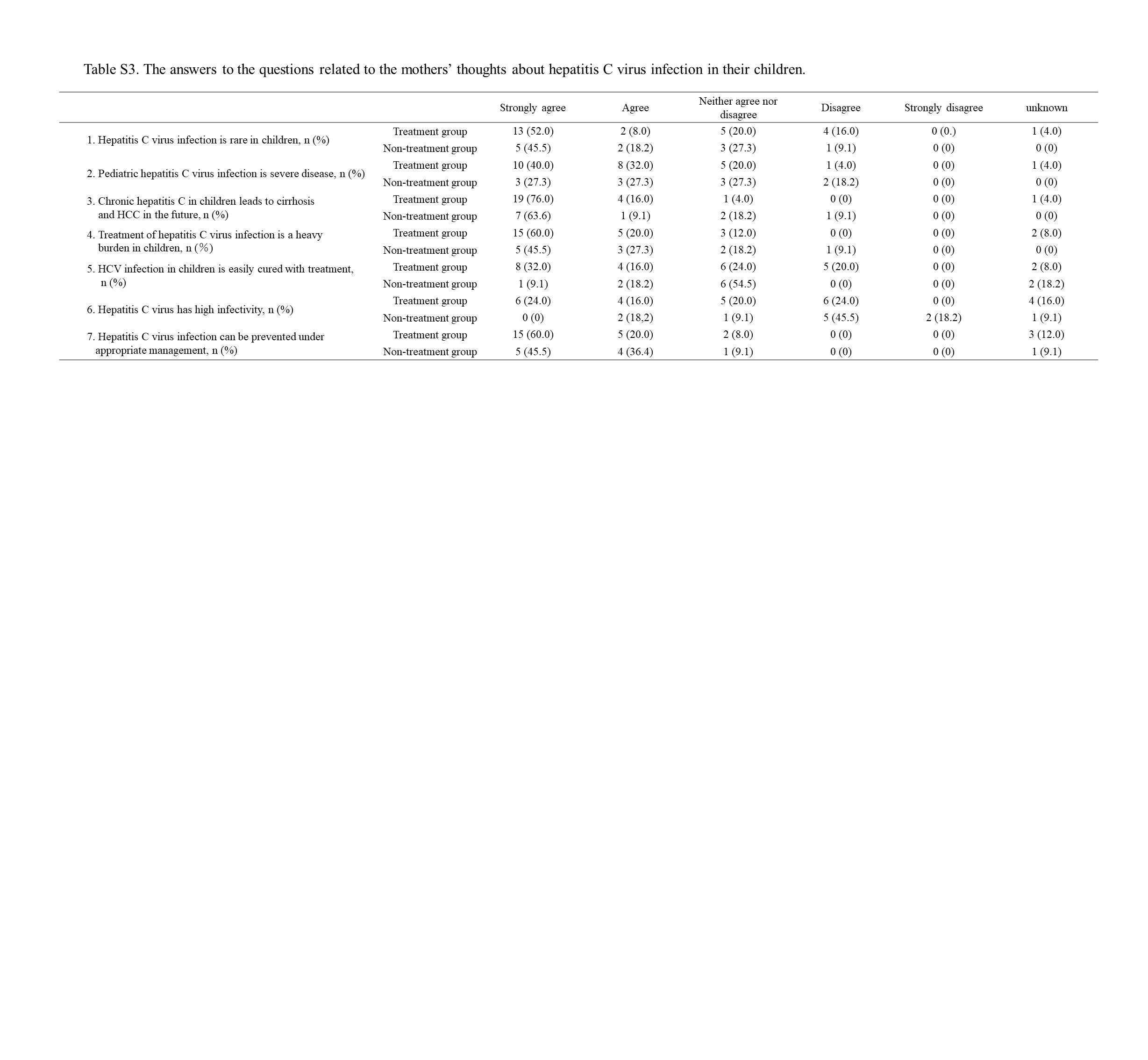

Supplement: Supplementary file 3 — Supplementary Table S3. [file 41598_2022_25519_MOESM3_ESM.jpg]
